# Rich Collection of n-Propylamine and Isopropylamine Conformers: Rotational Fingerprints and State-of-the-Art Quantum Chemical Investigation

**DOI:** 10.1021/acs.jpca.9b11767

**Published:** 2020-01-27

**Authors:** Mattia Melosso, Alessio Melli, Lorenzo Spada, Yang Zheng, Junhua Chen, Meng Li, Tao Lu, Gang Feng, Qian Gou, Luca Dore, Vincenzo Barone, Cristina Puzzarini

**Affiliations:** † Dipartimento di Chimica “Giacomo Ciamician”, Università di Bologna, Via Selmi 2, 40126 Bologna, Italy; ‡ Scuola Normale Superiore, Piazza dei Cavalieri 7, 56126 Pisa, Italy; § Department of Chemistry, School of Chemistry and Chemical Engineering, Chongqing University, Daxuecheng South Rd. 55, 401331 Chongqing, China

## Abstract

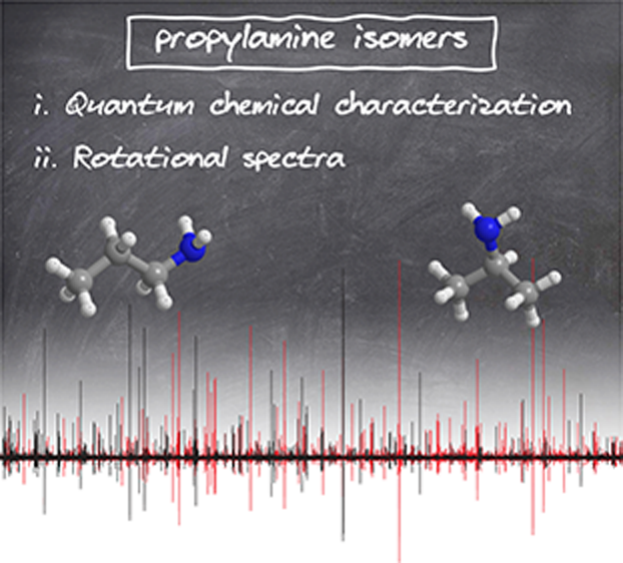

The conformational isomerism of isopropylamine and n-propylamine has been investigated by means of an integrated strategy combining high-level quantum-chemical calculations and high-resolution rotational spectroscopy. The equilibrium structures (and thus equilibrium rotational constants) as well as relative energies of all conformers have been computed using the so-called “cheap” composite scheme, which combines the coupled-cluster methodology with second-order Møller–Plesset perturbation theory for extrapolation to the complete basis set. Methods rooted in the density functional theory have been instead employed for computing spectroscopic parameters and for accounting for vibrational effects. Guided by quantum-chemical predictions, the rotational spectra of isopropylamine and n-propylamine have been investigated between 2 and 400 GHz with Fourier transform microwave and frequency-modulation millimeter/submillimeter spectrometers. Spectral assignments confirmed the presence of several conformers with comparable stability and pointed out possible Coriolis resonance effects between some of them.

## Introduction

Amines are widespread in nature and represent an important family of compounds involved in numerous natural and artificial mechanisms. The −NH_2_ moiety is found in several classes of compounds such as neurotransmitters, hormones, and alkaloids. Amines are also closely related to amino acids, from which they can be formed through decarboxylation reactions. These fundamental biological processes take place, for instance, during neurotransmitters formation. The reverse mechanism, namely amino acids formation, is of particular interest not only in biology but also in astrochemistry. Indeed, since the disproved detection of glycine,^1^ the astronomical identification of amino acids in the interstellar medium (ISM) has become a great challenge. It is therefore important to detect their potential precursors. In this respect, methylamine (CH_3_NH_2_) has been suggested to play an important role in the formation of glycine in the ISM.^2^ Similarly, more complex amines can be regarded as precursors of other proteinogenic amino acids.

Methylamine has already been observed in the ISM, toward the giant molecular cloud Sagittarius B2^3,4^ and in the hot cores associated with the high-mass star-forming region NGC 6334I.^5^ The abiotic formation of methylamine from ammonia (NH_3_) and methane (CH_4_) ices exposed to an ionizing radiation has been demonstrated in the laboratory and proposed as feasible in interstellar ices.^6^ In the same experiment, Förstel et al. also observed the formation of ethylamine (CH_3_CH_2_NH_2_) and suggested that more complex amines, for example, propylamine, can be produced with an increased CH_4_:NH_3_ ratio.^6^

Although the −NH_2_ moiety is found in some interstellar molecules (such as cyanamide,^7^ formamide,^8^ or aminoacetonitrile^9^), amines remain elusive species, methylamine being the only member of this family unequivocally identified in the ISM. Even ethylamine, the next member when increasing the alkyl chain length, has not conclusively been detected.^10^ Notwithstanding, even if a molecule is not identified, it is important to derive astronomical upper limits for its abundance to be compared with those of related species^11,12^ or within astrochemical models. In this context, laboratory studies and analyses of the rotational spectra of propylamine are mandatory for guiding future astronomical observations.

Besides its potential astrochemical interest, propylamine shows a rich conformational behavior. Starting from the two possible structural isomers, namely isopropylamine (IPA) and n-propylamine (PA), two and five stable conformers can exist, respectively. Because of such a structural variety, this molecule can be considered as a specimen for understanding, among other factors, how substituents affect the stability of isolated primary amines and consequently their chemical properties.

Here, we report on a joint spectroscopic and computational investigation of PA and IPA conformers, with the aim of providing an exhaustive spectroscopic characterization. The manuscript is organized as follows. First of all, the computational methodology and the experimental details are presented. Then the obtained results are described and discussed. Finally, concluding remarks will summarize the main outcomes of this work.

## Computational Details

A state-of-the-art computational investigation of iso- and n-propylamine has been carried out to provide a reliable guide to experiment. A preliminary study of the potential energy surface (PES) of both PA and IPA has been carried out at the B3LYP-D3(BJ)/SNSD level of theory^13,14^ (hereafter denoted as B3). To account for dispersion effects, Grimme’s DFT-D3 scheme^15^ in conjunction with the Becke-Johnson (BJ) damping function^16^ has been used. The SNSD (double- and triple-ζ basis sets of the SNS family are available for download at https://smart.sns.it) double-ζ basis set is obtained from the N07D basis set^17,18^ by inclusion of diffuse *s* functions on all atoms and one set of diffuse polarized functions (*d* functions on heavy atoms and *p* on H atoms).

A more accurate characterization of the stationary points of the PES has been next performed using a double-hybrid functional combined with a triple-ζ basis set, namely the B2PLYP-D3(BJ)/maug-cc-pVTZ-*d*H level^19,20^ (from here on referred to as B2). The basis set has been derived from the maug-cc-pVTZ one^21^ by removing the *d* functions on hydrogen atoms. The nature of the stationary points has been checked by evaluating and diagonalizing the Hessian matrix (second derivative of the energy in mass-weighted Cartesian coordinates).

The starting point for a spectroscopic characterization in the frame of rotational spectroscopy is the accurate determination of rotational constants. According to vibrational perturbation theory to second order,^22^ the vibrational ground-state rotational constants, *B*_0_^*i*^, can be expressed as
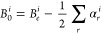
1where *i* denotes the principal inertia system axes (*a*, *b*, *c*), the sum running over all vibrational modes. *B*_*e*_^*i*^ values are the equilibrium rotational constants, which are straightforwardly obtained from the equilibrium structure, and the α values are the vibration–rotation interactions constants. Overall, 1/2∑_*r*_ α_*r*_^*i*^ denotes the vibrational correction to *B*_*e*_^*i*^ (*ΔB*_vib_). Since *B*_*e*_^*i*^ is significantly larger than *ΔB*_vib_, the computational effort is mainly put on the determination of the equilibrium geometry. To this purpose, the “cheap” composite scheme^23^ (shortly denoted as ChS) has been employed, whose formulation can be summarized as
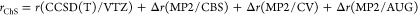
2where (i) the first term on the right-hand side is a generic structural parameter optimized at the fc-CCSD(T)/cc-pVTZ level of theory^24,25^ (“fc” denotes the frozen core approximation), where CCSD(T) stands for the coupled cluster (CC) singles and doubles approximation augmented by a perturbative treatment of triple excitations; (ii) the extrapolation to the complete basis set (CBS) limit is evaluated using Møller–Plesset perturbation theory to second order^26^ (MP2), thereby exploiting the *n*^–3^ formula by Helgaker et al.^27^ applied to the fc-MP2/cc-pVTZ and fc-MP2/cc-pVQZ optimized geometries; (iii) the third term introduces the core-correlation effect, which is estimated as difference between all-MP2/cc-pCVTZ^28^ and fc-MP2/cc-pCVTZ optimized parameters (“all” denoting the correlation of all electrons); and (iv) the last term accounts for the contribution of diffuse functions in the basis set, and it is evaluated as difference between fc-MP2/cc-pVTZ and fc-MP2/aug-cc-pVTZ^25,29^ optimized structural parameters. Even if the denomination “cheap” remarks its lower computational cost with respect to a full coupled-cluster approach, this scheme is expected to provide accurate results, with an estimated accuracy of 0.001–0.002 Å for bond lengths and 0.1–0.2° for angles.^23^

The evaluation of the second term on the right-hand side of eq 1, which contributes to *B*_0_^*i*^ values for about 1–3%,^30,31^ requires the calculation of an anharmonic force field. This can be evaluated at a lower level of theory, that is, the B3 level. To complete the spectroscopic characterization, quartic centrifugal distortion constants have been obtained from harmonic force field calculations, which have been performed at the B2 level. First-order properties, such as dipole moments and nuclear quadrupole coupling constants, have also been evaluated at the same level of theory. Finally, the sextic centrifugal distortion constants have been obtained as a byproduct of anharmonic force field calculations.

As far as the energetic characterization is concerned, the “cheap” composite scheme presented in eq 2 needs to be rearranged as follows:^23^
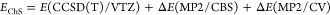
3In this formulation, analogously to eq 2, the first term on the right-hand side is the electronic energy calculated at the fc-CCSD(T)/cc-pVTZ level of theory. Instead, the second term is evaluated as sum of the HF/CBS and ΔMP2/CBS contributions, where the former is evaluated using the three-point formula by Feller^32^ applied to HF/cc-pVTZ, HF/cc-pVQZ, and HF/cc-pV5Z calculations. The ΔMP2/CBS term is the MP2 correlation energy extrapolated to the CBS limit using the formula proposed by Helgaker et al.,^27^ thereby employing the cc-pVTZ and cc-pVQZ bases. The last contribution of eq 3, *ΔE*(MP2/CV), is obtained analogously to the third term on the right-hand side of eq 2.

All DFT and MP2 calculations have been performed with the Gaussian16 suite,^33^ while all CCSD(T) computations were performed with the CFOUR package.^34^

## Experiment

Rotational spectra were recorded in Chongqing between 2 and 22 GHz with a Fourier transform microwave spectrometer (FTMW) and in Bologna in the range 80–400 GHz with a frequency-modulation millimeter-/submillimeter-wave (FMsubmm) spectrometer. Samples of PA (99%) and IPA (99%), both purchased from Adamas or Sigma-Aldrich, were used without any further purification.

In the FTMW experiment, a gas mixture of 1% PA (or IPA) in helium at a stagnation pressure of 2 bar was supersonically expanded through a solenoid valve (Parker-General Valve, Series 9, nozzle diameter 0.5 mm) into the Fabry-Pérot cavity of the spectrometer. Each transition is split into a Doppler pair because of the coaxially oriented beam-resonator arrangement (COBRA-type) of the supersonic jet. The line position is obtained as the arithmetic mean of the frequencies of the Doppler components. The estimated accuracy for frequency measurements is better than 3 kHz. Lines separated by more than 6 kHz are therefore resolvable.

The setup of the FM sub mm spectrometer, reaching frequencies as high as 1.6 THz, has been extensively described elsewhere.^35^ Briefly, a Gunn diode emitting in the W band (80–115 GHz) was used as primary source of radiation, whose frequency is stabilized by a Phase-Lock Loop (PLL) system and referenced to a 5 MHz rubidium atomic-clock. Passive multipliers (doubler and tripler) in cascade were used to reach higher frequencies. The output radiation was sine-wave modulated at 48 kHz and fed into the free-space glass absorption cell of the spectrometer, containing samples at a static pressure of 5 μ bar. The signal was detected by a Schottky barrier diode and demodulated by a Lock-in amplifier set at twice the modulation frequency (2*f*) so that the second derivative of the actual spectrum was displayed. An additional improvement of the signal-to-noise ratio of the spectrum is attained by filtering the Lock-in signal in a resistor-capacitor RC system.

### Isopropylamine

IPA is the branched isomer of propylamine that can exist in two conformational forms: *trans* and *gauche* (doubly degenerated), hereafter labeled as *T* and *G*, respectively. Both conformers have been identified in low-resolution spectroscopic studies reporting the Raman spectra of IPA in the gas/liquid/solid phases.^36,37^ Conversely, the rotational spectrum of IPA has been only observed for the most stable *trans* conformer.^38,39^ In the present work, the laboratory investigation of the rotational spectrum of *T*-IPA has been extended. We have also attempted to detect rotational features of *G*-IPA, but our observations could not be interpreted unambiguously.

**Figure 1:**
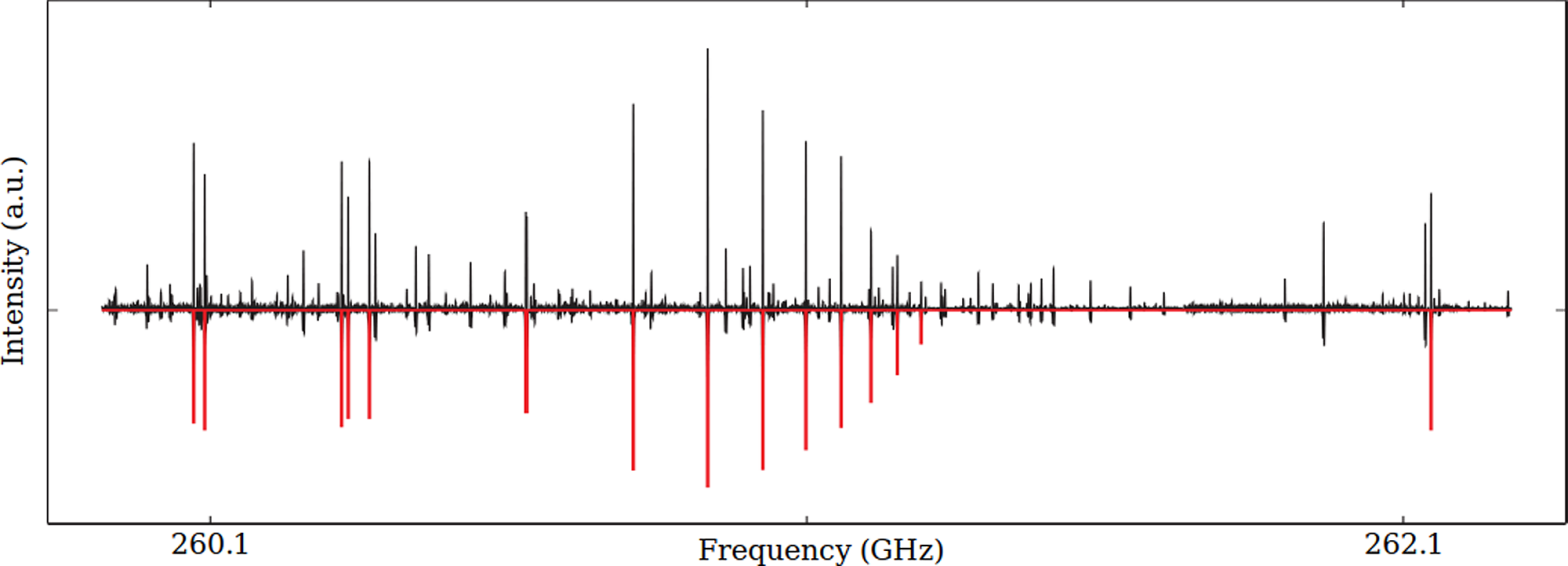
Portion of the millimeter-wave spectrum (black trace) of *T*-IPA (recording conditions: RC = 3 ms, FM = 300 kHz, *f* = 48 kHz, frequency step = 15 kHz, 400 s integration time). The red sticks represent the positions and the intensities of rotational transitions of *T*-IPA in the vibrational ground state.

The *trans* isomer is a nearly oblate asymmetric-top rotor (*k* = 0.81) belonging to the *C*_*s*_ point group. It possesses a permanent electric dipole moment μ = 1.19(3)D,^38^ lying almost completely on the *c* axis (μ_*c*_ = 1.19 ± 0.03 D and μ_*b*_ = 0.10 ± 0.04 D); μ_*a*_ is zero by symmetry. Therefore, the pure rotational spectrum of *T*-IPA is dominated by *c*-type transitions (see Figure 1), whereas *b*-type ones are expected to be ∼150-times weaker. The rotational energy levels of *T*-IPA can be described using Watson’s semirigid Hamiltonian,^40^ either in the *S*- or *A*-reduction. The latter reduced Hamiltonian has been employed both in quantum-chemical calculations and spectral analysis to facilitate the comparison between our results and those previously reported.^39^

The rotational Hamiltonian can be summarized as follows:
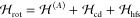
4where  contains the rotational constants in the Watson *A*-reduced form,^41^ the  part accounts for the centrifugal distortion terms (quartic, sextic, and so on), and  is the hyperfine-structure Hamiltonian due to the presence of nitrogen, whose nuclear spin is *I*_N_ = 1. The latter is responsible for a coupling between the nitrogen quadrupole moment with the electric field gradient at the nucleus, which determines a splitting of each rotational level in three sublevels according to the coupling scheme:
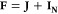
5Thus, the allowed sublevels correspond to the *F* values *J* + 1, *J*, *J* – 1; for *J*_*Ka*, *Kc*_ = 0_0,0_ only the *F* = 1 level exists.

For *T*-IPA, some rotational lines at frequencies lower than 50 GHz have already been measured with a Stark modulation spectrometer^38^ and a waveguide FTMW spectrometer.^39^ In both experiments, hyperfine splittings were resolved for many transitions. However, due to limited frequency and quantum number ranges, the resulting centrifugal analysis was not well-constrained, for example, the relative error on Δ_*K*_ was greater than 50%.

In the present work, the spectrum of *T*-IPA has been extended at higher frequencies (∼400 GHz), recording transitions between rotational energy levels with *J* and *K*_*c*_ quantum numbers as high as 30 and 20, respectively. The hyperfine structure due to nitrogen quadrupole coupling has been partially resolved for ten high-*K*_*c*_ transitions, while all remaining transitions (more than 150) have been observed as single lines. Moreover, some *b*-type transitions have been measured with the FTMW spectrometer, in the spectral region below 22 GHz. Despite their intrinsic low intensity, the favorable conditions of a supersonically expanded jet (rotational temperatures around a few Kelvin, no vibrational satellites) allowed us to record for the first time and confidently assign five *b*-type transitions of *T*-IPA. Although higher-frequency *b*-type transitions are expected to be well-predicted in terms of line positions, their weakness make them impossible to be unequivocally assigned in the submillimeter-wave spectrum.

The newly observed transitions of *T*-IPA, together with those reported in the literature,^38,39^ have been fitted to the Hamiltonian of eq 4 in a weighted least-squares procedure, as implemented in the SPFIT/SPCAT suite of programs.^42^ In the fitting procedure, each datum has been weighted proportionally to the inverse square of its uncertainty. The accuracy of our lines is estimated ranging between 10 and 20 kHz in the millimeter/submillimeter-wave regions (where linewidths are dominated by Doppler effect) and 3 kHz in the microwave region. Literature data have been given uncertainties depending on the residuals reported in the original papers, that is, 3 kHz for ref (39) and 100 kHz for ref (38). Since our spectral analysis is based on two different types of transitions, *b*- and *c*-type, most of the spectroscopic parameters could be obtained with satisfactory accuracy. The results are collected in Table 1 together with the computed values. The comparison between experiment and theory shows a good agreement. In particular, rotational constants agree within 0.1%. The uncertainties affecting the experimental rotational constants are smaller than 1 kHz, while the quartic centrifugal distortion terms are determined with relative errors smaller than 2% and in very good agreement with our *ab initio* values. Furthermore, five of the seven sextic centrifugal distortion parameters were refined in the least-squares procedure.

**Table 1:** Ground-State Spectroscopic Parameters (*A* Reduction, *III*^*l*^ Representation) of Isopropylamine

		*T*-IPA		*G*-IPA
parameter	unit	experimental	theoretical^a^	theoretical^a^
*A*	MHz	8331.90303(16)^b^	8321.69	8173.55
*B*	MHz	7977.33553(17)	7982.68	7958.44
*C*	MHz	4656.91658(63)	4652.23	4658.07
Δ_*J*_	kHz	7.16590(49)	7.26	7.02
Δ_*JK*_	kHz	–11.8645(18)	–12.1	–10.9
Δ_*K*_	kHz	5.64(13)	5.81	4.92
δ_*J*_	kHz	–0.149245(91)	–0.142	–0.120
δ_*K*_	kHz	9.2268(57)	9.40	7.48
Φ_*J*_	Hz	0.01132(52)	0.0113	–0.0362
Φ_*JK*_	Hz	–0.612(33)	–0.596	–0.406
Φ_*KJ*_	Hz	1.93(10)	1.88	1.50
Φ_*K*_	kHz		–1.30	–1.06
ϕ_*J*_	mHz		–2.21	–1.94
ϕ_*JK*_	Hz	0.0765(74)	0.095	0.0324
ϕ_*K*_	Hz	–6.71(39)	–6.73	–8.32
χ_*aa*_	MHz	1.7890(19)	2.06	–3.39
χ_*bb*_	MHz	2.5688(18)	2.68	2.24
|μ_*a*_|	D		0.00	1.15
|μ_*b*_|	D	0.10(4)	0.07	0.36
|μ_*c*_|	D	1.19(3)	1.30	0.40
No. data		235		
*J*_max_, *K*_c max_		30, 20		
*rms* error	kHz	14.0		
σ		0.95		

a ChS equilibrium rotational constants augmented by vibrational corrections at the B3 level. Quartic centrifugal distortion and nuclear quadrupole coupling constants as well as dipole moment components at the B2 level. Sextic centrifugal distortion constants at the B3 level.

b Values in parentheses denote one standard deviation and apply to the last digits of the constants.

**Figure 2:**
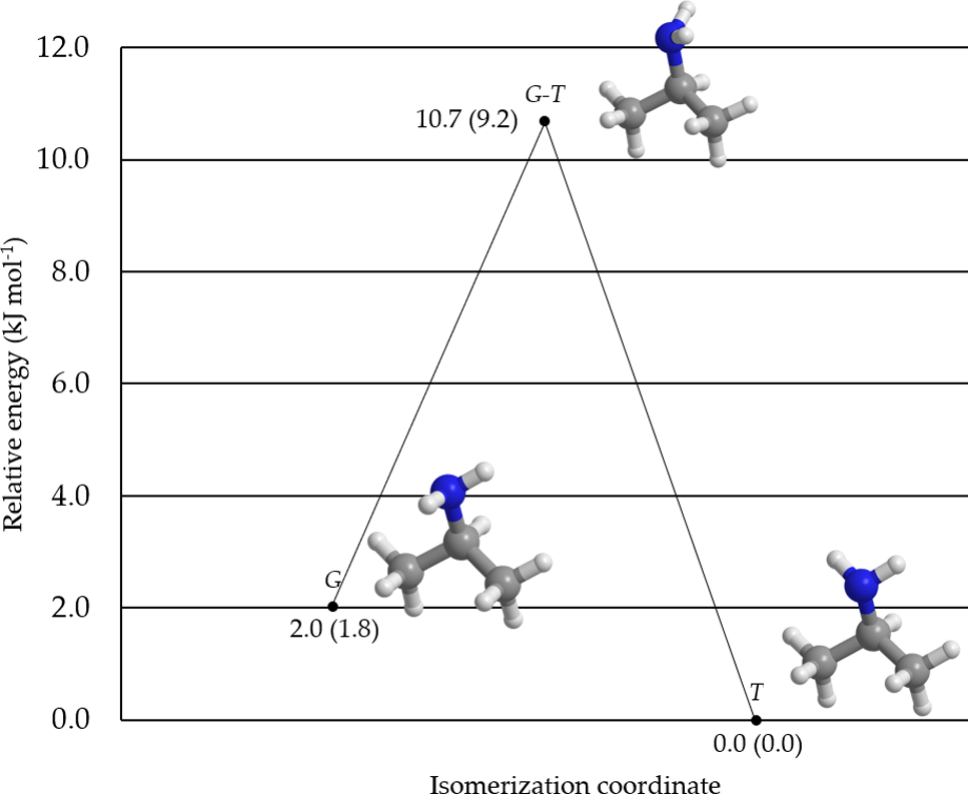
Schematic representation of the relative ChS energies of the IPA system at the B2 geometry, including the conformational transition state. The ChS energies augmented by the harmonic ZPE correction at the B2 level are reported within parentheses. All values are in kJ mol^–1^.

Table 1 also summarizes the spectroscopic parameters computed for *G*-IPA, for which the rotational spectrum could not be assigned confidently despite the fact that the energy difference between *G*- and *T*-IPA is estimated to be lower than 2 kJ mol^–1^ and the conformers are separated by a conformational barrier of ∼10 kJ mol^–1^ (see Figure 2). Indeed, the high barrier and the small energy difference should favor the spectral observation of both conformers. However, there are different factors that could prevent the detection of *G*-IPA rotational signatures. First, *G*-IPA is a nearly oblate asymmetric-top rotor with a medium-intensity *a*-type spectrum. The combination of these two issues results in a sparse rotational spectrum without any characteristic pattern. From a visual inspection, it is therefore hard to correctly assign *G*-IPA lines in the spectrum. Second, other small amines have been suggested to exist in both *trans* and *gauche* forms by *ab initio* calculations or low-resolution experiments (e.g., see refs (43, 44) for aminoacetonitrile and propargylamine), but only their *trans* forms (i.e., the most stable ones) have been revealed by high-resolution molecular spectroscopy.^45,46^

### n-Propylamine

PA is the linear isomer of propylamine with the NH_2_ group at one end of the carbon chain. Compared to IPA, it shows an increased flexibility and thus a greater number of stable conformers. The conformational behavior of PA has already been the subject of low-resolution Raman and infrared investigations,^47−49^ which pointed out the presence of five different conformers with relative energies within 2 kJ mol^–1^. On the other hand, the rotational spectrum of PA has never been studied to date. To establish the structures and energetics of the possible conformers of PA, we have combined extensive *ab initio* calculations with high-resolution laboratory spectra.

**Figure 3:**
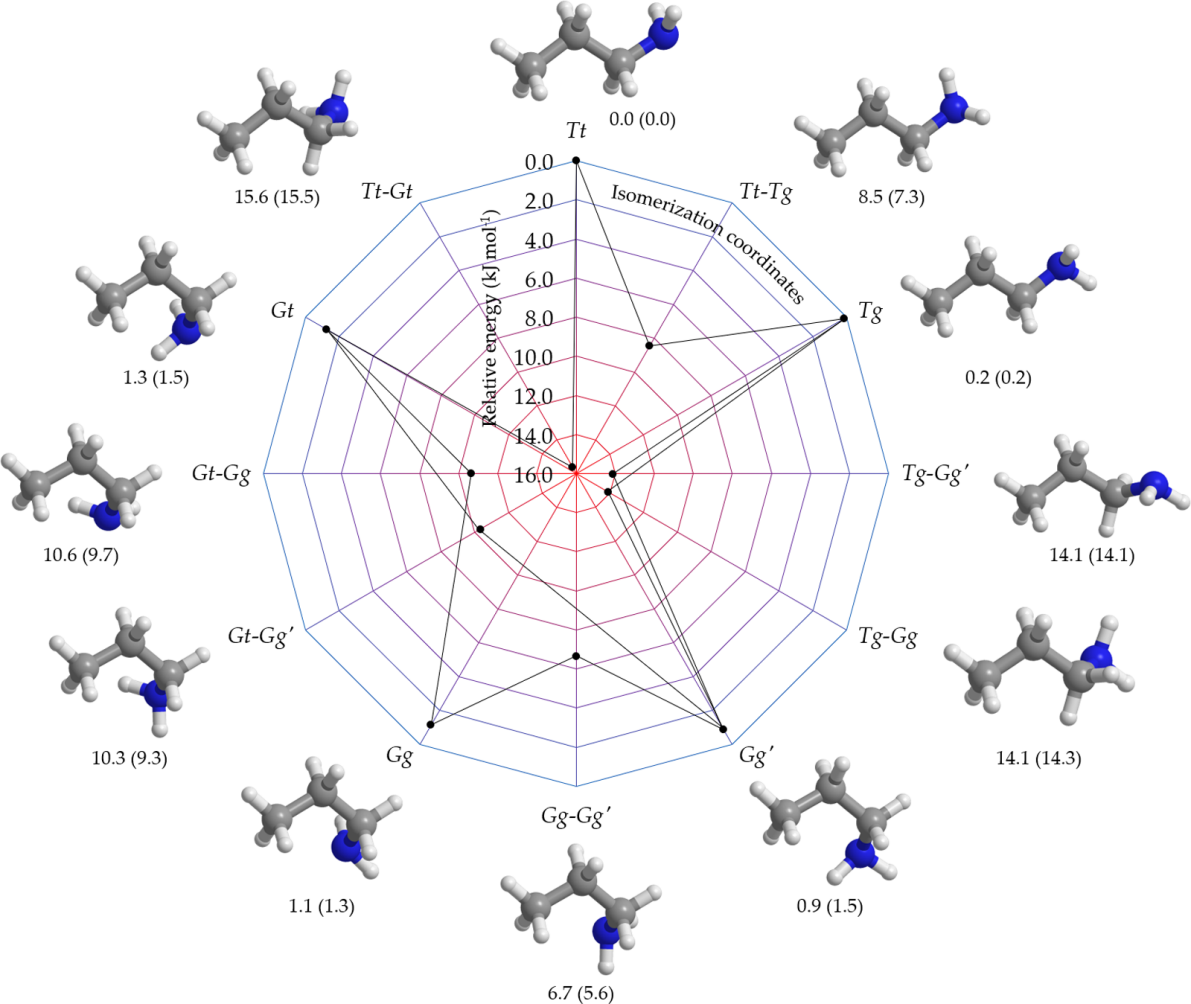
Schematic representation of the relative ChS energies of the PA system at the B2 geometry including the conformational transition states. The ChS energies augmented by the harmonic ZPE correction at the B2 level are reported within parentheses. All values are expressed in kJ mol^–1^ with respect to the *Tt* isomer.

The relative energies of the five possible conformers, namely *Tt*, *Tg*, *Gg*, *Gg*′, and *Gt*, have been computed as described in the Computational Details section. Our results, which also include the conformational transition states, are graphically displayed in Figure 3. In the labeling of the conformers, the capital letter refers to the conformation of the NC–CC dihedral angle (*T* standing for *trans* and *G* for *gauche*) and the lower-case letter refers to the value of the :N–CC dihedral angle. For the latter, starting from the *t* (*trans*) position, clockwise and counterclockwise rotations of the NH_2_ group by 120 degrees along the N–C bond are indicated by *g* and *g*′, respectively. It is evident from Figure 3 that the *Tt* conformer is the most stable one, followed by *Tg* (0.2 kJ mol^–1^). All *Gauche* conformers have energies within 2 kJ mol^–1^: 1.3 kJ mol^–1^ for *Gt*, 1.1 kJ mol^–1^ for *Gg*, and 0.9 kJ mol^–1^ for *Gg*′. As a result, the room temperature population of PA is spread over all conformers, which are expected to be abundant enough to be observed in the spectrum. Given that, we have predicted the rotational spectra of each conformer on the basis of our best computed spectroscopic parameters (see Tables 2 and 3).

**Table 2:** Ground-State Spectroscopic Parameters (*S* Reduction, *I*^r^ Representation) of *trans* Conformers of n-Propylamine

		*Tt*-PA		*Tg*-PA
parameter	unit	experimental	theoretical^a^	theoretical^a^
*A*	MHz	24633.518(12)^b^	24616.75	25043.54
*B*	MHz	3687.54437(15)	3690.50	3732.78
*C*	MHz	3473.87413(14)	3477.17	3491.10
*D*_*J*_	kHz	0.815768(21)	0.815	0.821
*D*_*JK*_	kHz	–2.12254(59)	–2.31	–2.56
*D*_*K*_	kHz	47.81(72)	49.2	51.82
*d*_1_	kHz	–0.066047(21)	–0.0664	–0.0779
*d*_2_	Hz	0.6651(65)	0.371	–1.87
*H*_*J*_	mHz		0.176	0.173
*H*_*JK*_	Hz		0.000156	–0.00112
*H*_*KJ*_	Hz	–0.0679(28)	–0.0744	–0.0873
*H*_*K*_	Hz		0.279	0.273
*h*_1_	mHz		0.0449	0.0428
*h*_2_	μHz		0.133	3.29
*h*_3_	μHz		–0.349	0.0694
χ_*aa*_	MHz	–0.4251(24)	–0.50	2.68
χ_*bb*_	MHz	–1.3669(31)	–1.58	0.13
|μ_*a*_|	D	Y^c^	0.98	0.05
|μ_*b*_|	D	Y	1.05	0.67
|μ_*c*_|	D	N	0.00	1.06
No. data		213		
*J*_max_, *K*_a max_		47, 17		
*rms* error	kHz	10.4		
σ		0.83		

a ChS equilibrium rotational constants augmented by vibrational corrections at the B3 level. Quartic centrifugal distortion and nuclear quadrupole coupling constants as well as dipole moment components at the B2 level. Sextic centrifugal distortion constants at the B3 level.

b Values in parentheses denote one standard deviation and apply to the last digits of the constants.

c For each μ_*i*_, “Y” and “N” refer to detected and nondetected *i*-type transitions, respectively.

**Table 3:** Ground-State Spectroscopic Parameters (*S* Reduction, *I*^r^ Representation) Determined for *Gauche* Conformers of n-Propylamine

		*Gt*-PA		*Gg*-PA		*Gg*′-PA	
constant	unit	exp.	theo.^a^	exp.	theo.^a^	exp.	theo.^a^
*A*	MHz	13760.8904(15)^b^	13784.02	13901.5751(12)	13997.20	13844.2441(13)	13894.36
*B*	MHz	4873.8863(11)	4818.57	4908.50289(71)	4866.42	4994.9267(12)	4975.73
*C*	MHz	4149.3176(15)	4108.95	4217.56546(60)	4203.29	4186.2431(13)	4179.34
*D*_*J*_	kHz	4.246(69)	4.32	4.415(35)	4.29	4.461(37)	4.68
*D*_*JK*_	kHz	–17.53(26)	–18.6	–16.81(14)	–17.4	–19.57(27)	–19.9
*D*_*K*_	kHz		58.8		58.3		57.7
*d*_1_	kHz	–1.546(56)	–1.29	–1.161(25)	–1.27	–1.603(42)	–1.48
*d*_2_	kHz		–0.0947		–0.100		–0.114
χ_*aa*_	MHz	–3.1427(27)	–3.36	0.2350(19)	0.27	1.9959(29)	2.26
χ_*bb*_	MHz	2.3210(31)	2.46	–1.9533(20)	–2.24	2.3372(34)	2.51
|μ_*a*_|	D	Y^c^	1.27	Y	0.78	N	0.07
|μ_*b*_|	D	N	0.05	Y	1.01	Y	0.08
|μ_*c*_|	D	Y	0.61	Y	0.25	Y	1.23
No. data		35		49		26	
*J*_max_, *K*_a max_		3, 1		3, 1		4, 2	
*rms* error	kHz	3.0		3.9		3.5	
σ		1.00		1.28		1.18	

a ChS equilibrium rotational constants augmented by vibrational corrections at the B3 level. Quartic centrifugal distortion and nuclear quadrupole coupling constants as well as dipole moment components at the B2 level.

b Number in parentheses are one standard deviation in units of the last quoted digit.

c For each μ_*i*_, “Y” and “N” refer to detected and nondetected *i*-type transitions, respectively.

PA is a nearly prolate asymmetric-top rotor (*k* = −0.98 for *T* conformers, −0.85 for *G* ones) with a total permanent dipole moment ranging between 1.2 and 2.0 D, depending on the conformer under consideration. Only *Tt*-PA is of *C*_*s*_ symmetry, while all the other conformers belong to the *C*_1_ point group. The rotational energy levels of PA can be described using an Hamiltonian analogous to that of eq 4. In this case, the *S*-reduced Watson Hamiltonian was used instead.^50^ Since there were no previous works for comparison, the rotational transitions of PA have been fitted to the *S*-reduced form of the Watson Hamiltonian, which usually requires a lower number of centrifugal distortion terms for a converged fit.

The spectral assignment procedure started at low frequencies, with the FTMW experiment. Even though the supersonic jet is “cold”, the room temperature population of the various PA conformers is maintained inside the Fabry-Pérot cavity of spectrometer because of the high barriers that separate the minima (see Figure 3). The rotational spectra of four conformers of PA could be assigned, mainly relying on the computed rotational and nuclear quadrupole coupling constants. Regrettably, the *Tg* conformer was not detected and a tentative explanation will be given later in the text.

Once the first rotational lines had been assigned and the spectroscopic parameters refined, it was possible to investigate the PA rotational spectrum at higher frequencies with the FMsubmm spectrometer. For the *Tt* conformer, the spectrum has been recorded and assigned up to 310 GHz. Both *a*- and *b*-type transitions have been detected with similar intensities, in accordance with the predicted dipole moment components. On the contrary, no μ_*c*_-allowed transitions were observable for symmetry reason. Thanks to the analysis of more than 200 lines for *Tt*-PA, it was possible to accurately determine the values of the rotational constants, all quartic and one sextic (*H*_*KJ*_) centrifugal distortion terms, and the nuclear quadrupole constants. As in the case of IPA, the experimental uncertainty of each transition frequency was estimated to be 3 kHz for FTMW data and 10–20 kHz for millimeter/submillimeter-wave lines, and the least-squares procedure was performed using the SPFIT/SPCAT program. The results of the fit are collected in Table 2, where the computed spectroscopic parameters of *Tt*-PA and *Tg*-PA are also reported. The agreement between experimental and theoretical values is excellent, with particular emphasis on the rotational constants for which the discrepancies are below 0.1%. A good agreement is also noted for the nuclear quadrupole constants and quartic centrifugal terms, the only exception being *d*_2_, whose experimental value is nearly twice the computed one. Furthermore, the quality of our fit is demonstrated by the low standard deviation (σ = 0.83) and *rms* error (10 kHz); these results provide a valid indication that the Hamiltonian appropriately describes the *Tt*-PA conformer.

The situation is different for the four remaining PA conformers. Although the *Gauche* family of conformers was easily identified in the FTMW spectrum, their rotational lines at millimeter-wavelengths seemed to deviate from the semirigid rotor approximation. This fact is not surprising, as it has already been observed in similar systems, for example, n-propanol (n-prOH, hereafter). The rotational spectrum of n-prOH has been studied with broadband spectroscopy from 8 to 550 GHz and resonance effects due to Coriolis interactions between the various conformers were recognized.^51^ Indeed, the five possible conformers of n-prOH (*Tt*, *Tg*, *Gg*, *Gg*′, and *Gt*, as for PA) are close in energy and they can be coupled through the Coriolis operators. This results in a perturbation of the rotational energy levels, which are no longer reproduced by the semirigid Hamiltonian of eq 4. A more appropriate Hamiltonian has first been developed for ethanol^52^ and successively applied to n-prOH (see eqs 1–3 of ref (51)). However, it is beyond the scope of this paper to perform a thorough analysis of the Coriolis interactions present in the ro-vibrational manifolds of PA, and we therefore limit ourselves to discuss the assignment of the low-frequency spectra.

**Figure 4:**
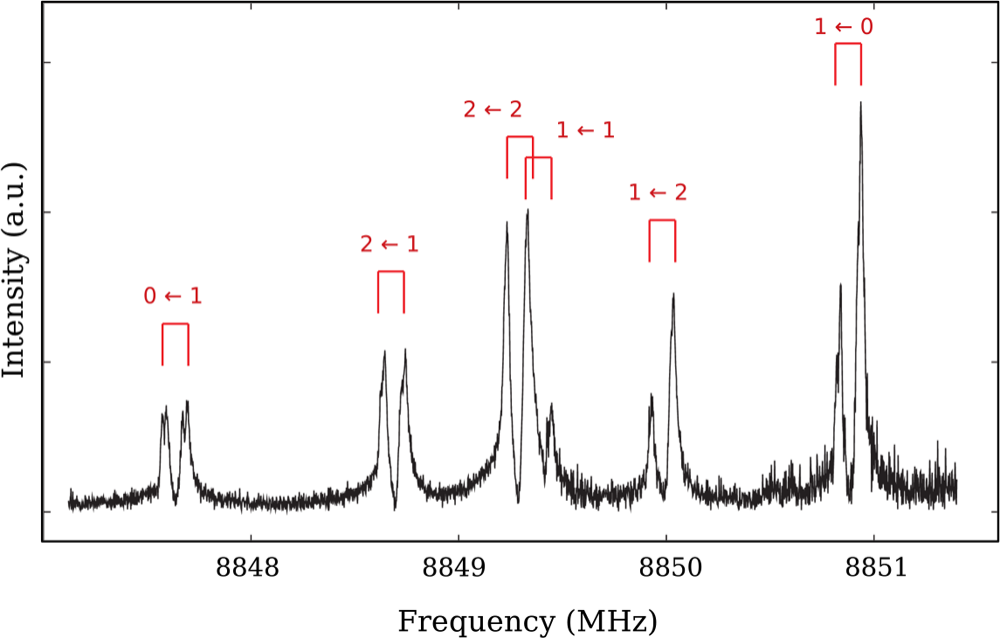
Hyperfine structure of the *J*_*Ka*,*Kc*_ = 1_1,1_ ← 1_0,1_ transition of *Gg*′-PA. Doppler splittings due to the coaxial arrangement of the FTMW spectrometer are shown in red for each component, together with the change of the quantum number *F*.

As already anticipated, all *Gauche* conformers of PA have been observed in the FTMW spectrum by assigning their lowest *J* transitions. As an example, the *c*-type 1_1,1_ ← 1_0,1_ transition of *Gg*′-PA is shown in Figure 4. Few tens of lines were recorded and analyzed for each conformers, thus allowing the determination of the rotational constants, some quartic centrifugal distortion terms and nuclear quadrupole coupling constants. These results are reported in Table 3, together with the corresponding computed counterparts. Upon inspection of this table, our theoretical values are in very good agreement with those experimentally determined.

The refined sets of spectroscopic parameters of Table 3 represent good starting points for searching rotational lines of the *Gauche* conformers at higher frequencies. To some extent, spectral lines above 80 GHz are easily assignable to the correct rotational transitions on the basis of predicted line-frequency and intensity. However, even if most of the lines are only few MHz off from spectral predictions, their inclusion in the fit leads to anomalous centrifugal distortion parameters, and the data are no longer reproduced within their experimental accuracy. These are plain evidence of the aforementioned perturbation effects.

As far as the *Tg*-PA conformer is concerned, its nondetection in the FTMW spectrum may appear a little puzzling. However, if the PA system is assumed to behave like the n-prOH system, the perturbation of the *Tg* conformer is expected to be considerably greater than that in the *Gauche* species and to occur even at the lowest *J* levels.^51^ Such perturbation effects could also explain the anomalous value of *d*_2_ obtained for *Tt*-PA.

## Conclusions

The conformational isomerism of two primary amines with chemical formula C_3_H_9_N, namely isopropylamine and n-propylamine, has been investigated by state-of-the-art quantum-chemical calculations and high-resolution molecular spectroscopy. The “cheap” composite scheme^23^ has been successfully applied to the evaluation of energetics and equilibrium structures of several stable conformers, namely *T*- and *G*-IPA as well as *Tt*-, *Tg*-, *Gg*-, *Gg*′-, and *Gt*-PA. The ChS method has been indeed designed to obtain accurate equilibrium geometries even for flexible systems.^53^ This has been confirmed by the present investigation: the very good agreement between experiment and theory for rotational and nuclear quadrupole coupling constants points out the accuracy and reliability of the ChS structures.

Furthermore, the presence of five different conformers with close relative stability makes PA a good test case for the hybrid CC/DFT methodology. The experiment has demonstrated that ChS equilibrium rotational constants combined with reliable vibrational corrections at the B3 level can lead to very accurate predictions of experimental ground-state rotational constants. The agreement obtained is indeed remarkable, the discrepancies being, in relative terms, of the order of 0.1%.

Rotational spectra have been recorded for most of the IPA and PA conformers, and the observed transitions could be easily assigned on the basis of our computed spectroscopic parameters. Particularly, the rotational spectra of *T*-IPA and *Tt*-PA (i.e., the most stable conformers of the two isomeric forms) have been investigated from the microwave region to the submillimeter-wave domain and satisfactorily reproduced with experimental accuracy. Some perturbation effects, likely due to Coriolis resonances, have been recognized in the spectra of the *Gauche* conformers of PA. Also, tentative explanations for the nondetection of *G*-IPA and *Tg*-PA have been given.

As a future perspective, we note that a broadband spectrum would be of great help to (i) take into account Coriolis couplings between different *Gauche*-PA conformers and (ii) detect the elusive *G*-IPA and *Tg*-PA isomers, thus allowing for an exhaustive characterization of the conformational behavior of IPA and PA. Moreover, the robust sets of spectroscopic constants determined in this work for *T*-IPA and *Tt*-PA are capable to provide reliable spectral predictions for rotational transitions up to 500 GHz, thus enabling dedicated astronomical searches of propylamine isomers.

## Supporting Information Available

List of observed transitions and their residuals from the final fit; optimized geometries at B2 level for all stationary points and ChS geometries for minima (ZIP)
